# Visual Rounds Based on Multiorgan Point-of-Care Ultrasound in the ICU

**DOI:** 10.3389/fmed.2022.869958

**Published:** 2022-05-25

**Authors:** Jia-Yu Mao, Hong-Min Zhang, Da-Wei Liu, Xiao-Ting Wang

**Affiliations:** ^1^Department of Critical Care Medicine, Peking Union Medical College Hospital, Peking Union Medical College and Chinese Academy of Medical Science, Beijing, China; ^2^Department of Health Care, Peking Union Medical College Hospital, Peking Union Medical College and Chinese Academy of Medical Sciences, Beijing, China

**Keywords:** point-of-care ultrasonography, visual round, intensive care unit, multiorgan, clinical practice

## Abstract

Point-of-care ultrasonography (POCUS) is performed by a treating clinician at the patient's bedside, provides a acquisition, interpretation, and immediate clinical integration based on ultrasonographic imaging. The use of POCUS is not limited to one specialty, protocol, or organ system. POCUS provides the treating clinician with real-time diagnostic and monitoring information. Visual rounds based on multiorgan POCUS act as an initiative to improve clinical practice in the Intensive Care Unit and are urgently needed as part of routine clinical practice.

## Introduction

Patient care rounds are a central part of daily clinical routine and play a vital role in quality management in intensive care. Point-of-care ultrasonography (POCUS), an emerging tool performed by frontline clinicians caring for critically ill patients especially in the intensive care unit (ICU), has been strongly supported by a growing body of evidence in several disciplines (1). POCUS could rapidly and accurately access their patients' clinical status to guide therapy, especially in circulatory and respiratory failure. Multiorgan POCUS further combines organ functions such as the renal, gastrointestinal, and neurologic systems (2). Visual rounds based on multiorgan POCUS act as an initiative to improve clinical practice and are urgently needed for routine clinical care. We will describe in this review the use of POCUS as part of the daily round in the intensive care unit, and will highlight the emerging use of visual rounds based on multiorgan POCUS as an initiative to improve clinical practice in the ICU.

## Materials and Methods

A literature search of Pubmed, Medline, Embase, Scopus and Cochrane library databases was conducted for the period Jan 1st 2000–2022 to identify all publications on point of care ultrasound in critical ill adult patients, using English language restriction, and the following MeSH query: [“point of care ultrasound” OR (“lung ultrasound”) OR “echocardiography” OR “cardiac ultrasound” OR “renal ultrasound” OR “gastrointestinal ultrasound” OR “abdominal ultrasound” OR “brain ultrasound” OR “neurological ultrasound”] AND [“critical care”]. Non-pertinent findings were discarded. Duplicates were removed. This strategy identified 873 records (Supplementary File 1).

### Critical Care Ward Round

Critical care is always facing complex, life-threatening conditions and needs to make rapid decision on account of incomplete data. Patient care rounds in the ICU include discussions based on reviewing clinical data and concluding care plans (3). Rounds are a crucial portion in daily clinical schedule in ICU. During patient care rounds, essential diagnostic, therapeutic and organizational affairs of patient care should be discussed in a structured form (4). The critical cognition round ranged from basic clinical scenarios and professional courses to a multidisciplinary round. Rounds play a vital impact on patient safety and quality management in the ICU (5).

### Point-of-Care Ultrasonography

POCUS is ubiquitous in critical care and advantages on its real-time and absence of radiation. POCUS has quickly emerged as a tool used by frontline clinicians caring for critically ill patients. It enables quickly identifying and guiding the management of conditions with hemodynamic instability or respiratory failure and is the standard of care for procedural guidance (6). POCUS developed from early identification to assessment admission to visual rounds, finally building a critical thinking system. Currently, more and more evidence strongly supports the significance of POCUS in numerous fields, especially highlighted in the management of COVID-19 (7, 8). Although respiratory abnormities are the most common performance, other organ systems may also be affected by COVID-19. POCUS is particularly appealing because it is not only rapid, bedside, and goal-oriented but also allows for the imaging of the cardiovascular, respiratory, abdominal, renal and neurological system (Table 1).

**Table 1:** Main indications and limits of POCUS in clinical practice.

**POCUS**	**Application**	**Limit**
Critical care echocardiography	➢ Fluid status assessment and management ➢ Assessment of ventricular function ➢ Assessment of organic abnormality	➢ Indicators interpretation should take into account of influencing factors ➢ Preexisting disease should be recognized first
Lung Ultrasonography	➢ Respiratory failure diagnosis ➢ Respiratory support strategies, like mechanical ventilation guidance, prone positioning and ECMO support ➢ Ventilation weaning	➢ Automated and quantitative analysis is awaiting to be improved ➢ Lesions adjacent to the pleural surface are more sensitive
Diaphragm ultrasound	➢ Monitoring ventilation-induced diaphragmatic injury ➢ Assessment of weaning or extubation failure	➢ Diaphragmatic weakness and injury still need further studied and verified
Renal ultrasound	➢ Detection and adjustment of systemic hypoperfusion ➢ Assessment of renal morphological anomaly or obstruction ➢ Evaluation of microcirculation through contrast -enhanced ultrasonography	➢ Further study was needed to explore correlation of indicators with disease and response to treatment ➢ Image is easily affected by respiration
Gastrointestinal ultrasound	➢ Monitoring gastrointestinal dysfunction ➢ Ultrasound-guided jejunal nutrition tube placement ➢ Identifying cause of acute abdomen	➢ Image quality is easily influenced by air-filled bowel or obesity
Neurological ultrasound	➢ Intracranial pressure monitoring ➢ Intracranial vascular flow monitoring	➢ ICP consists of several components and could only be partially reflected by the according indicators

### Visual Rounds Based on Multiorgan POCUS

#### Cardiovascular System

**Figure 1:**
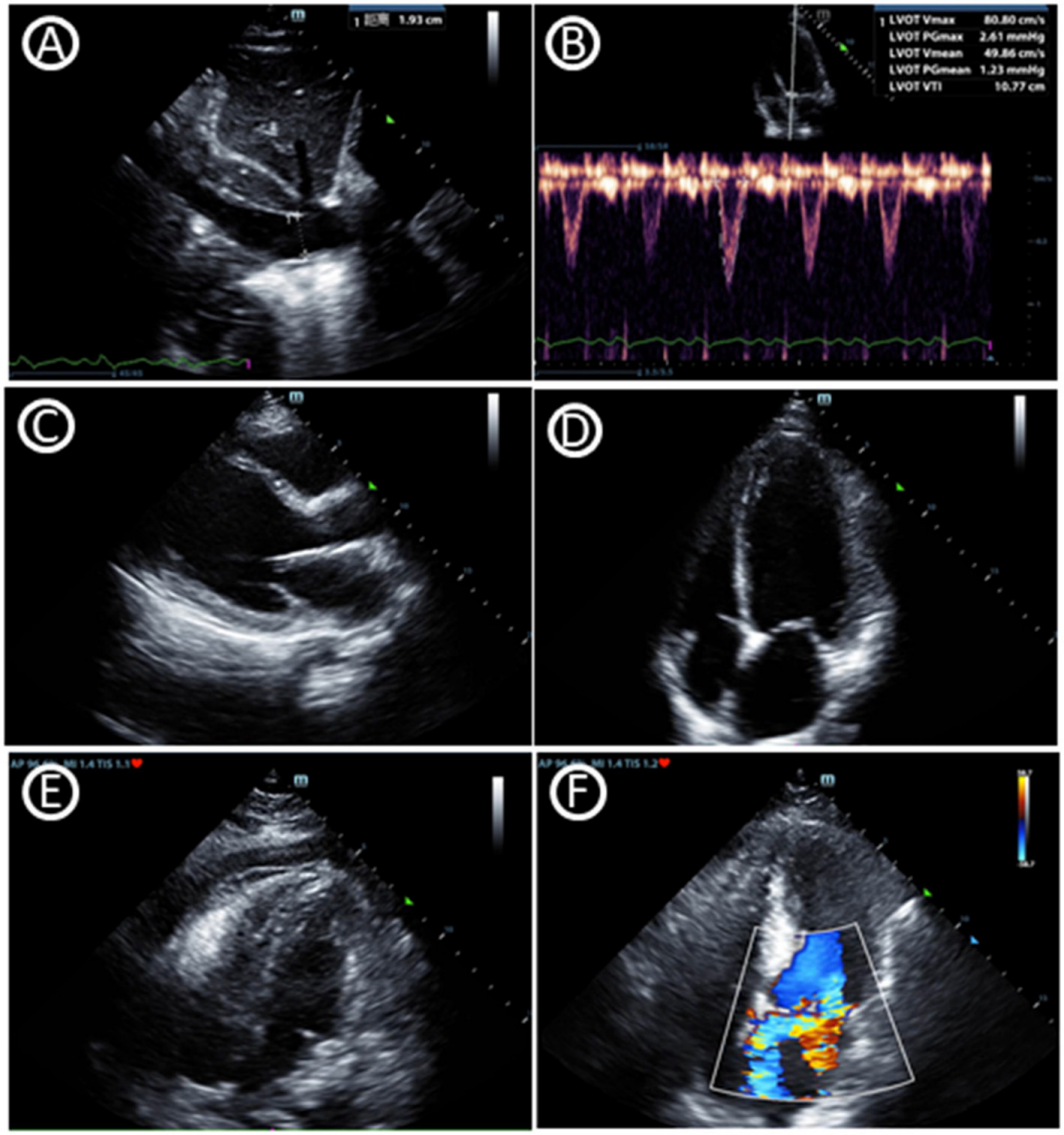
Critical care echocardiography imaging. **(A)** Inferior vena cava diameter. **(B)** Velocity-time integral of left ventricular outflow tract, which can help to gain cardiac output and fluid responsiveness. **(C)** Parasternal window parasternal long-axis view. **(D)** Apical window four-chamber view. **(E)** Pericardial effusion in subcostal window four-chamber view. **(F)** Mitral regurgitation in apical window four-chamber view.

POCUS is not only a diagnostic tool but also helps making clinical decision in real time at the bedside. Critical care echocardiography (CCE) (9), including transthoracic echocardiography (TTE) and transesophageal echocardiography (TEE), facilitates the correct classification of shock (10, 11). *1. Volume Status:* A perpetual challenge observed daily in the ICU is estimating volume status and assessing their potential of cardiac output augmentation with the infusion of intravascular volume. The respiratory variation of the inferior vena cava has been widely used to determine volume responsiveness, which should also be interpreted combining the patient's underlying pathophysiological status like the mode of ventilation/respiration, paitient‘s respiratory pattern, cardiac conditions and other factors like increased abdominal pressure (12). Variables based on stroke volume or cardiac output were also combined with other factors to predict flood responsiveness (13). *2. Ventricular Function:* Assessment of left ventricular (LV) systolic function (as well as cardiac output), LV diastolic function (left atrial pressure) and right ventricular dysfunction (pulmonary hypertension) are skills required for CCE. LV systolic dysfunction, based on the regions involved, could be divided into regional wall motion abnormality (RWMA) and diffuse hypokinesia. *3. Organic Abnormality:* Assessment of valvular stenosis and regurgitation are also skilled by CCE through the application of pulsed wave and continuous wave Doppler. Intensivists with CCE knowledge could assess evidence of pericardial effusion, even cardiac tamponade, which are common reasons of obstructive shock. Left ventricular outflow tract obstruction (LVOTO) can also be easily diagnosed through CCE which is often underestimated in ICU patients (Figure 1).

Overall, CCE could efficiently assist in early shock diagnosis, typically categorized into hypovolemic, cardiogenic, obstructive, and distributive shock (14). Based on the instructions of CCE, intensivists could rapidly and accurately assess their patients' clinical state to guide therapy, such as fluid resuscitation, the application of inotropes, and even pericardiocentesis in cardiac tamponade or the use of thrombolytics for massive pulmonary embolism. Therefore, an increasing number of comprehensive reviews support use of CCE in the management of hemodynamic failure (10, 11, 15, 16). Vignon et al. also conducted a prospective multicenter study comparing hemodynamic assessment in sepsis patients through CCE and invasive transpulmonary thermodilution, the agreement was moderate to good (17). Moreover, the accuracy of critical care TEE was also confirmed compared with TEE or TTE performed by cardiologists (18). All of the above findings indicate that intensivists performing CCE should be a standard constitution of daily practice for critical care. However, some affairs should be taken into consideration when interpreting signs of CCE. A variety of abnormal signs found in critically ill patients could be not only the causes of hemodynamic instability, but also the consequences of preexisting cardiac diseases (19). What‘s more, CCE may also be influence by other factors like the respiratory state. For example, the respiratory variation of the inferior vena cava, which reflects circulatory capacity and cardiac function, is also related with respiratory effort and intrathoracic pressure.

#### Respiratory System

**Figure 2:**
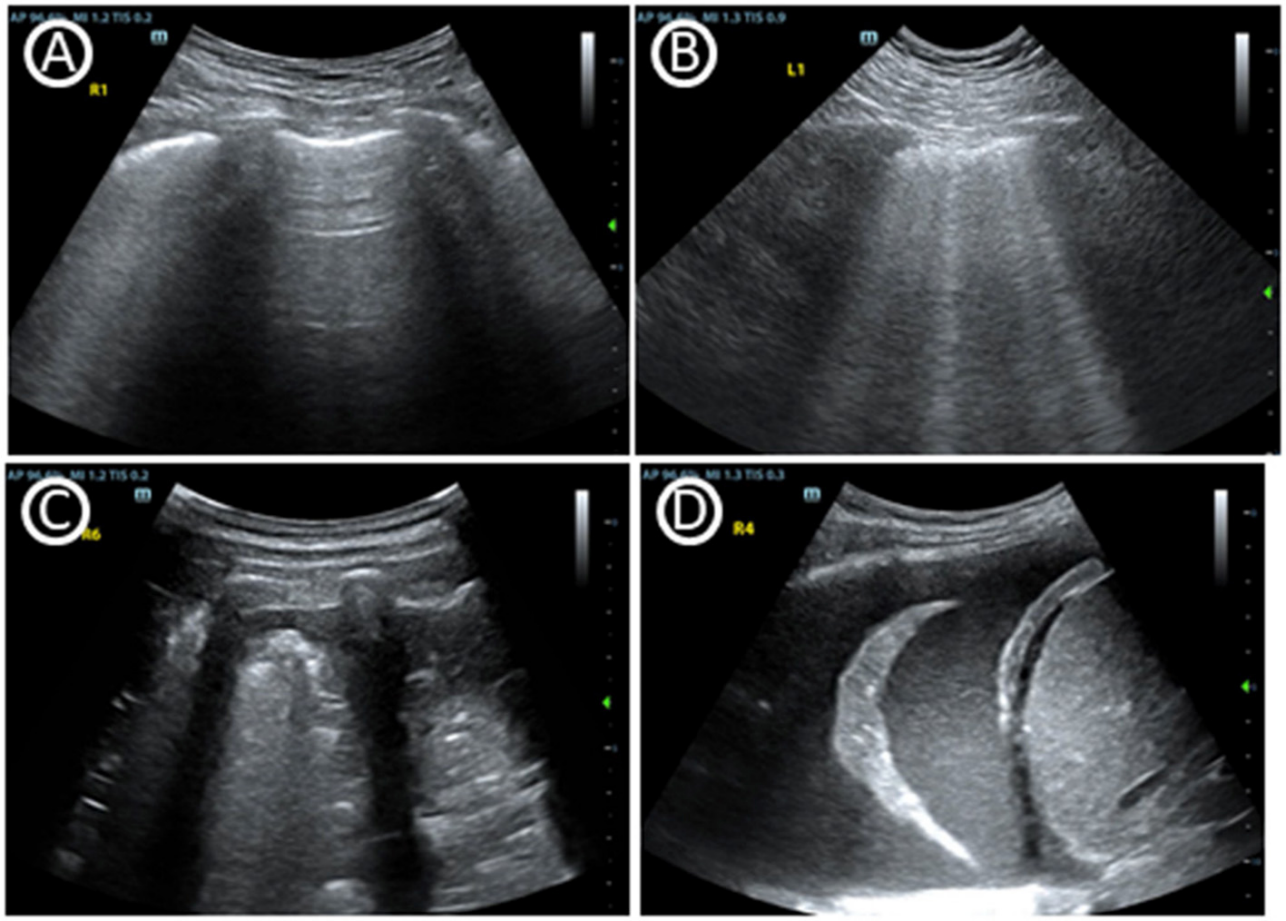
Basic signs of lung ultrasound. **(A)** A-lines, horizontal reverberation artifacts at a regular distance indicate a high gas–volume ratio below the parietal pleura. **(B)** Multiple B lines, are vertical artifacts deriving from the pleural line, moving synchronously with lung sliding, usually reaching the bottom of the screen and erasing A-lines. **(C)** Tissue-like pattern identifies lobar consolidation. **(D)** Pleural effusion.

Although the use of lung ultrasonography (LUS) in critical care demonstrated by Dr. Daniel Lichtenstein in the 1990s (20), it has been especially highlighted in the management of COVID-19 in recent years (8). LUS is skilled in identifying lung conditions based on the constitution of air and fluid inside (Figure 2). *1. Pneumothorax:* As a differential diagnosis for dyspnea in the ICU, pneumothoraces occur episodically. The presence of a “lung pulse” may exclude pneumothorax with 100% specificity and 66% sensitivity (21, 22). The presence of lung sliding may also exclude pneumothorax with a 100% negative predictive value (23, 24). *2. Interstitial Syndrome:* B-lines in LUS are a type of comet-tail artifact, which are characterized in the interstitial syndrome. B-lines root from thickened interlobular septae, however the clinician must identify its etiology from fibrosis, pulmonary edema or inflammation. *3. Consolidation:* Consolidation is easily accessed by LUS since lung tissue is airless. Clinician also need to identify its etiology from atelectasis, pneumonia and even pulmonary infarct. *4. Pleural Effusion:* LUS can easily detect pleural effusion and provide more information on fluid characteristics. For instance, COVID-19-associated pneumonia present with heterogeneous B-line, irregular pleura and consolidations similar as other viral pneumonia (25).

As a worldwide effort to spread POCUS as a basic skill in ICU, LUS is outstanding for a wide variety of lung conditions. *1. Repiratory failure diagnosis:* As is shown above, LUS is skilled in identifying lung conditions based on the constitution of air and fluid inside, to distinguish phenmothorax, interstitial syndrome, consolidation or pleural effusion. LUS has shown to be superior in evaluating patients with dyspnea (26), especially in detecting pulmonary congestion in heart failure (27, 28). LUS was recommened for patients with acute dyspnea especially by American College of Physicians and Society of Critical Care Medicine (Table 2) (29, 30). LUS is an invaluable tool in the intensivist's armamentarium as a valuable alternative to CXR or CT (31, 32). Quantitative LUS analysis was superior in the assessment of extravascular lung water than CT, which is advantage in physical density (33). LUS is also outstanding in diagnosis and management of acute respiratory distress syndrome (ARDS). ARDS can be easily confirmed by LUS through the recognition of a typical pattern characterized by B-lines, spared areas, pleural line thickening, and subpleural consolidations (34). LUS in conjunction with CCE could help to detect not only cardiogenic pulmonary edema but also acute pulmonary embolism (PE). *2. respiratory support strategies:* In addition to establishing a diagnosis of acute dyspnea, LUS could also assist in respiratory support strategies (35). LUS has the advantages of predicting response to recruitment (36), prone positioning (37, 38), assessment of extravascular lung water and fluid management (39, 40), and even extracorporeal membrane oxygenation (ECMO) support (41). Combined with CCE, RV function is also important to be evaluated in the ventilation strategy and management in ARDS. *3.Ventilation weaning: Diaphragmatic ultrasound* including diaphragmatic thickness, its fraction and mobility may be a valid tool (42). LUS combining with CCE and diaphragm ultrasound may effectively reduce ventilation-induced diaphragmatic injury (43, 44) and extubation failure (44–47). Diaphragmatic weakness and spontaneous respiration induced diaphragmatic injury are still need further studied and verified. LUS is a rapid and convenient imaging technology that has important applications for daily use in the ICU.

**Table 2:** Main recommendation in society guidelines for POCUS.

**Guideline**	**Evidence**	**Recommendation**
2021 ACP	Conditional recommendation; low-certainty evidence	Use point-of-care ultrasonography in addition to the standard diagnostic pathway when there is diagnostic uncertainty in patients with acute dyspnea
2020 EFSUMB		Gastrointestinal ultrasound in intestinal emergencies, including bowel obstruction, gastrointestinal perforation and acute ischemic bowel disease
2019 ASE		Guidelines for the performance of a comprehensive transthoracic echocardiographic examination
2015 SCC	Strong recommendation, evidence A strong recommendation, evidence B	Diagnosis of pleural effusion; Diagnosis of pneumothorax; Preload responsiveness, ventilated; Pulmonary hypertension; Ventricular tachycardia/fibrillation; Cardiac tamponade; Shock, undifferentiated; Prosthetic valve endocarditis; Blunt chest trauma for pericardium; transesophageal echocardiography

However, LUS is limited since it‘s only sensitive with lung lesions adjacent to the pleural surface. Although LUS provides relevant information on morphological and functional changes occurring in the lungs, it still need to be automated to reduce inter- and intra-observer variability. Visual LUS score could indicate pulmonary edema, but it‘s poorly correlated with pulmonary capillary wedge pressure and extravascular lung water at high PEEP (48). LUS could help score PEEP-induced lung recruitment, however it could not assess PEEP-induced hyperinflation which need to combine other imagine tools (49). In addition, use of LUS should take into account patient population and clinical conditions. For example, the sensitivity of LUS in the FAST exam in pediatric trauma might not be as reliable as in adults (50). In the initial evaluation of chest trauma, using LUS as the primary imaging modality should be considered with caution (51).

#### Renal Ultrasound

**Figure 3:**
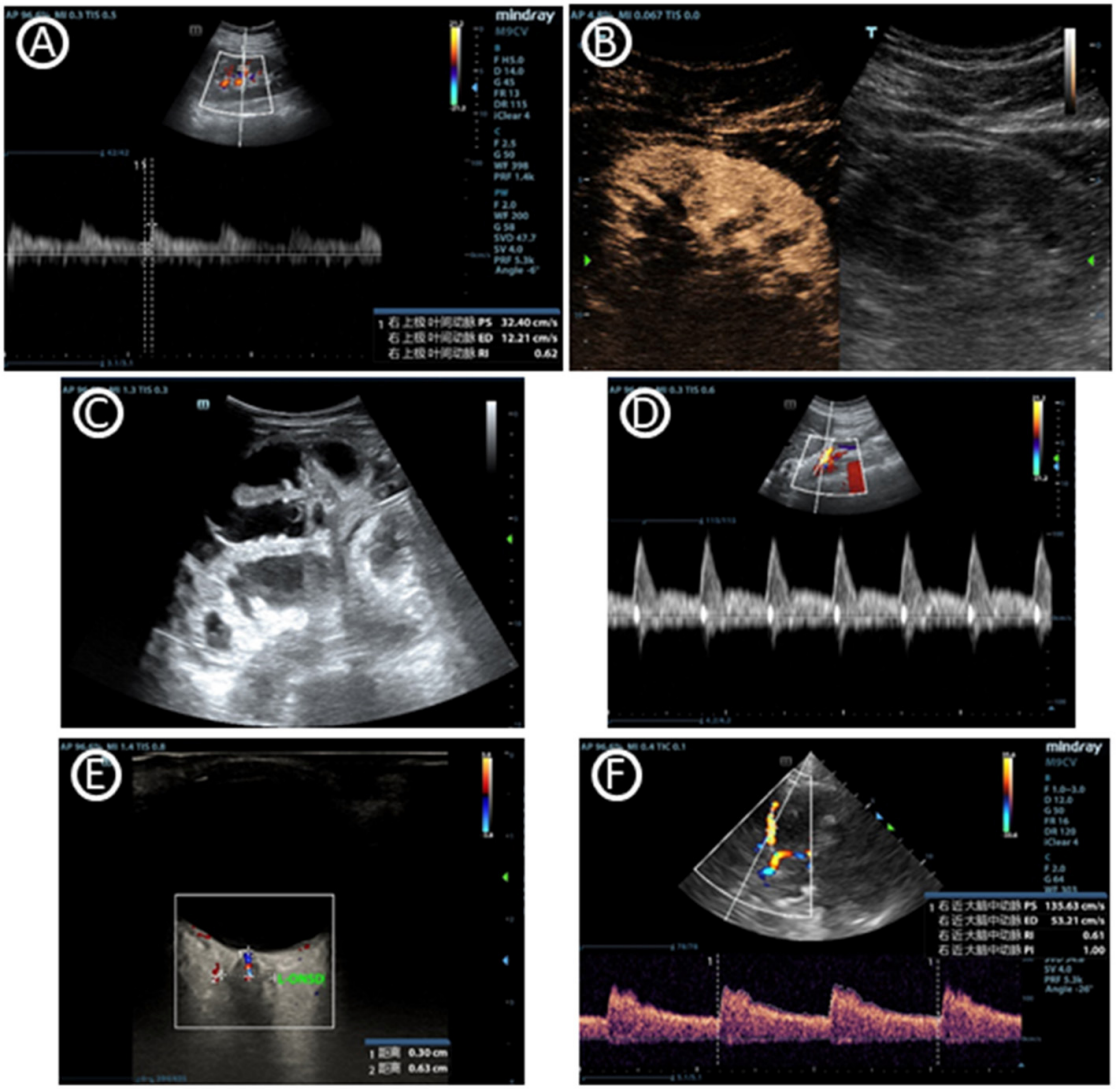
Images of multiorgan ultrasound. **(A)** Normal doppler tracing of the interlobar vessels in renal ultrasound. The renal resistive index can be measured using the maximal and the minimal arterial velocity during the cardiac cycle. **(B)** Renal contrast-enhanced ultrasonography. **(C)** Dilated fluid-filled bowel loops that indicates small bowel obstruction. **(D)** Blood flow curve of superior mesenteric vein. **(E)** Optic nerve sheath diameter, which can help to predict intracranial pressure. **(F)** Cerebral blood flow velocity of middle cerebral artery accessed by transcranial doppler ultrasonography.

Acute kidney injury (AKI), shown as oliguria or elevated serum blood urea nitrogen or creatinine, is common in ICU. Renal ultrasound may help clinician to define the circumstances bedside (Figure 3) (52, 53). The CCE that was mentioned previously could guide shock differentiation and volume status assessment. Renal ultrasound could evaluate renal size, echogenicity, vascularity, and the presence of urinary tract obstruction, imaging both kidneys and the bladder. Resistive index (RI) of the arcuate and interlobar arteries was widely studied as a potential objective marker for renal pathology, and was shown to be a prognostic factor (54, 55). RI showed advantage in detecting hypoperfusion due to hypovolemia and hemodynamics titration to improve renal blood flow (56). However, although RI has been studied extensively, its interpretation remains particularly complex and is highly dependent on the clinical setting (57). Renal contrast-enhanced ultrasonography can be used to non-invasively evaluate renal macrocirculation and microcirculation in critical ill patients. Decrease in renal blood flow, particularly cortical blood flow, may be observed in septic AKI through the technology and may contribute to its development (58).

#### Gastrointestinal Ultrasound

In addition to the Focused Assessment with Sonography in Trauma protocol (FAST) used in trauma patients, there is an additional focus on gastrointestinal (GI) failure or dysfunction that is evaluated by ultrasound (59–61). There is currently a lack of universally accepted criteria for gastrointestinal failure or dysfunction in critical care. Gastrointestinal ultrasound can provide its anatomical and functional information through the lumen, wall and the surrounding structures of the stomach and bowel. Evaluation of functional processes like peristalsis and blood flow may be used in combination (Figure 3) (62). Monitoring GI function through gastrointestinal ultrasound may effectively assess acute gastrointestinal injury, predict feeding intolerance and lead to appropriate therapeutic interventions. An interdisciplinary group of European experts also summarizes use of gastrointestinal ultrasound in identifying causes of acute abdomen like bowel obstruction, gastrointestinal perforation and acute ischemic bowel disease (Table 2) (63). Just like other ultrasound types, adequate training is needed to use and interpret the ultrasound images correctly. In gastrointestinal ultrasound, image quality was easily influenced by air-filled bowel or obesity.

#### Neurological Ultrasound

In the clinical management of several neurological diseases, intracranial pressure (ICP) is an important monitoring indicator. Since ICP is not conveniently monitored, non-invasive monitoring of ICP (nICP) may be helpful to improve the clinical management of these conditions. Cerebral blood flow velocity (FV) accessed by Transcranial Doppler ultrasonography (TCD) is an effective technique (64, 65). The optic nerve sheath diameter measurement may also reveal that a congested papilla develops with optic disc elevation when the increase in ICP persists (Figure 3) (66, 67). Neurological ultrasound combing with LUS also helps when intracranial hypertension and severe lung damage coexist in the same clinical scenario, since recommending ventilation strategies in ARDS may potentially increase intracranial hypertension. Combined use of LUS with optic nerve sheath diameter assessment and TCD can contribute to a tailored brain-protective ventilation strategy (68). However, some studies showed TCD may errorly affect clinical decision-making. Perhaps it is because of the different elastic modulus of different intracranial tissues that ICP cannot be measured like the liquid pressure in a closed container. ICP monitoring based on TCD is a potential research direction, but its clinical value is still further verified.

### Perspectives and New Applications of POCUS

Key trends and future applications in POCUS technology may include handheld ultrasound system and artificial intelligence. *Handheld ultrasound systems* based on the telemedicine technology is becoming available to the frontline clinician (69). The number of probes necessary affects its clinical utility. *Artificial intelligence* based on quantitative analysis is advancing. Tele-ICU, which promoting the use of telemedicine in critically ill patients, has been vigorously development in recent years (70, 71). The use of telecommunications has become the prototype for telemedicine in health care practice and has been described since the advent of telecommunication. Imaging and visual rounds are of increasing importance. New prospects for use of POCUS not only as a diagnostic but also as a monitoring tool are approaching. Automatic and quantitative scoring system was developed to integrate and standardize the clinical assessment of the lung (72, 73). Classification of benchmark ultrasound images is required for training a convolutional artificial network (74). The 5G-powered robot-assisted teleultrasound diagnostic system has already been applied in intensive care units (75), which has an advantage as easy operation, good feasibility, comparable diagnosis, and deserves to promote widely in clinical practice.

However, the lack of standardized therapeutic plans based on POCUS results makes it‘s difficult to study the effect of POCUS on patient outcomes (1). What‘s more, the clinician using POCUS must be competent in it, POCUS training poses a great challenge as it is developing rapidly. The training curricula and methods to assess competence is imperative for the safe and effective use of such systems (76).

## Conclusion

Recognization and estimation of critical manifestation is the principal objective in constructing a therapeutic plan. POCUS is now increasingly comprehensive and used in predicting pathogenesis and guiding multiorgan function monitoring. Circulatory and respiratory failure is the major concern of critical scenarios, POCUS is not only advantaged in analysis of this, but also combining organ functions such as the renal, gastrointestinal, and neurologic systems. POCUS is now recognized as a fundamental component of critical care. The wide range of applications of POCUS includes the evaluation of the critical cause as well as therapy monitoring. Future development on handheld ultrasound system and artificial intelligence point toward POCUS as a standard tool of the frontline clinician. Overall, the clearly delineated competencies of POCUS has been recommended for critical care fellowship training for more than a decade. Visual rounds based on multiorgan POCUS in the ICU are an urgently needed part of routine clinical care.

## Author Contributions

D-WL designed the experiment. J-YM drafted and revised the manuscript. H-MZ and X-TW conceived of the study, participated in its design of the study. All authors have read the manuscript and approved of the version to be published.

## Funding

This work was supported by grants from the Natural Science Foundation of Beijing Municipality (No. 7194306).

## Conflict of Interest

The authors declare that the research was conducted in the absence of any commercial or financial relationships that could be construed as a potential conflict of interest.

## Publisher's Note

All claims expressed in this article are solely those of the authors and do not necessarily represent those of their affiliated organizations, or those of the publisher, the editors and the reviewers. Any product that may be evaluated in this article, or claim that may be made by its manufacturer, is not guaranteed or endorsed by the publisher.

## Abbreviations

POCUS: point-of-care ultrasonography
ICU: intensive care unit
CCE: critical care echocardiography
TTE: transthoracic echocardiography
TEE: transesophageal echocardiography
LV: left ventricular
RWMA: regional wall motion abnormality
LVOTO: left ventricular outflow tract obstruction
LUS: lung ultrasonography
CT: computed tomography
CXR: chest x-ray
ARDS: acute respiratory distress syndrome
ECMO: extracorporeal membrane oxygenation
PE: pulmonary embolism
AKI: acute kidney injury
RI: resistive index
FAST: Focused Assessment with Sonography in Trauma
GI: gastrointestinal
ICP: intracranial pressure
FV: flow velocity
TCD: Transcranial Doppler Ultrasonography.
