# Supplementary material for: Visual Rounds Based on Multiorgan Point-of-Care Ultrasound in the ICU
Source: Front Med (Lausanne). 2022 May 25;9:869958. doi: 10.3389/fmed.2022.869958 (PMC9174546; doi:10.3389/fmed.2022.869958)
Supplement: Supplementary file 1 [file Data_Sheet_1.PDF]

PRISMA 2020 flow diagram for new systematic reviews which included searches of databases and registers only

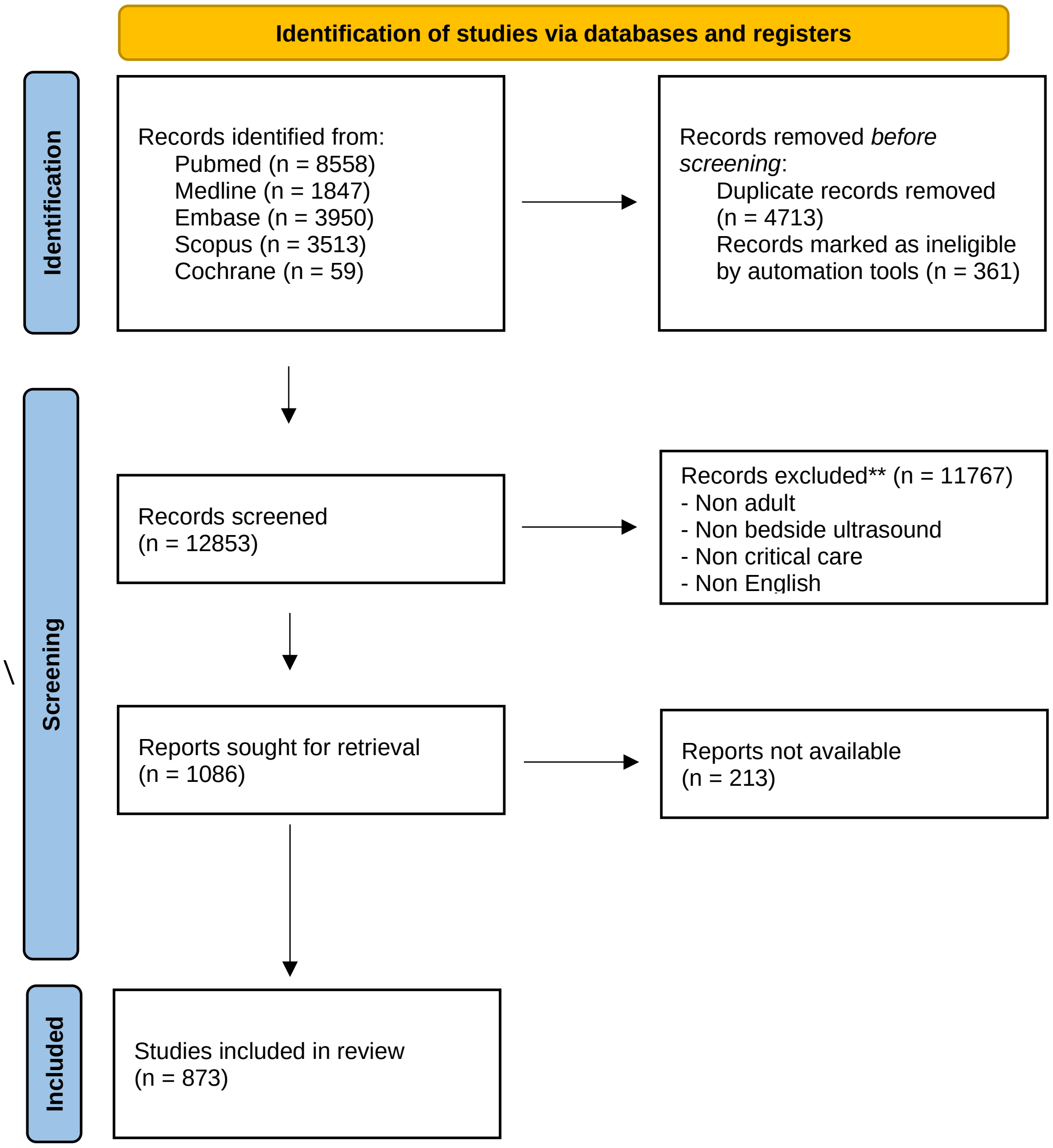

From: Page MJ, McKenzie JE, Bossuyt PM, Boutron I, Hoffmann TC, Mulrow CD, et al. The PRISMA 2020 statement: an updated guideline for reporting systematic reviews. BMJ 2021;372:n71. doi: 10.1136/bmj.n71

For more information, visit: http://www.prisma-statement.org/
